# Co‐cultivation and Medium Optimization: A Strategy for Discovering Fungal‐derived Protease Inhibitors

**DOI:** 10.1002/cbdv.202501135

**Published:** 2025-09-02

**Authors:** Vitor de Souza Mazucato, Ludmilla Tonani, Marcia Regina von Zeska Kress, Adriano Defini Andricopulo, Gisele Barbosa, Renata Krogh, Leonardo Luiz Gomes Ferreira, Paulo Cezar Vieira

**Affiliations:** ^1^ Department of BioMolecular Sciences, Ribeirão Preto School of Pharmaceutical Sciences University of São Paulo Ribeirao Preto Brazil; ^2^ Department of Clinical Analysis, Toxicology, and Food Science Ribeirão Preto School of Pharmaceutical Sciences University of São Paulo Ribeirao Preto Brazil; ^3^ São Carlos Institute of Physics, University of São Paulo Sao Carlos Brazil

**Keywords:** antifungal activity, cysteine protease inhibitors, fungal co‐cultivation, *Fusarium*, *Trypanosoma cruzi*

## Abstract

The fungi *Fusarium guttiforme* and *Phytophthora palmivora* were cultivated in four different media (Potato Dextrose Agar, Czapek, rice, and ISP2) and co‐cultured to stimulate fungal interactions and enhance secondary metabolite production. Promising extracts were fractionated, yielding compounds such as the iron complex of fusaric acid (**1**), magnesium complex of fusaric acid (**2**), haematocin (**4**), fusarinolic acid (**7**), and cyclonerodiol (**8**). These compounds exhibited significant papain inhibitory activity, with compounds **2** and **4** showing IC_50_ values below 20 µM. Notable anti‐*Trypanosoma cruzi* activity was also observed, particularly for compounds **2** (50% inhibitory concentration [IC_50_] = 12.19 µM) and **4** (IC_50_ = 13.32 µM). Additionally, antifungal activity against *Candida* spp. was detected, with compound **2** and the magnesium complex of 9,10‐dehydrofusaric acid complex (**3**) showing MICs of 50 µg/mL. This study highlights the potential of fungal co‐cultivation and variation in culture medium as effective strategies for discovering novel bioactive protease inhibitors and antiparasitic agents.

## Graphical abstract

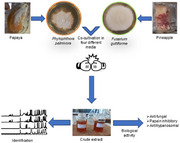

## 1 Introduction

Fungi represent a significant source of bioactive metabolites, widely recognized for their chemical diversity and potential to produce compounds with diverse chemical structures [1]. Since the successful isolation of penicillin from the fungus *Penicillium rubens* (previously named P. notatum and, until recently, *P. chrysogenum*) by Alexander Fleming in 1928 and later developed by Howard Florey, numerous studies have explored the potential of fungi in the discovery of biologically active natural compounds. Over the decades, countless therapeutic agents have been developed based on fungal metabolites [2].

Among modern approaches to maximize the biosynthetic potential of fungi, the “One Strain Many Compounds” (OSMAC) approach stands out. This method aims to activate silent biosynthetic pathways (gene clusters) under standard cultivation conditions, enabling the production of a broader array of secondary metabolites from a single fungal strain [3, 4]. Practical application of this approach includes altering culture conditions, such as carbon and nitrogen sources or the addition of metabolic precursors, which can result in significantly different metabolic profiles [5, 6].

Another promising approach is co‐cultivation, which involves the simultaneous cultivation of different fungal species. This strategy simulates natural interactions, such as mutualism or antagonism, which induce competition for nutrients and space. Such interactions may stimulate the activation of secondary metabolic pathways, resulting in the biosynthesis of new bioactive compounds or increased production of known metabolites [4, 7, 8]. For instance, the co‐cultivation of *Fusarium guttiforme* with *Colletotrichum horii* in Potato Dextrose Broth (PDB) led to the production of novel compounds, including derivatives of fusaric acid [9].

The discovery of novel compounds is crucial for disease treatment, particularly in developing effective antifungal agents against resistant strains. Pathogens such as *Candida* spp., responsible for over 1.6 million deaths annually, underscore the need for innovative therapeutic molecules [10, 11, 12].

Beyond fungi, proteases are also promising therapeutic targets due to their central role in biological processes, including programmed cell death, protein degradation, and extracellular matrix remodeling [13, 14, 15]. In protozoans, these enzymes are essential for parasite survival. While *Trypanosoma cruzi* relies on cruzain, *Leishmania* spp. depend on cysteine proteases CPA, CPB, and CPC during their life cycle [15, 16, 17, 18, 19]. Classified into 14 clans and 82 families, the most abundant cysteine proteases share structural homology with papain. These enzymes, known as PLCPs, belong to the CA clan and play key roles in virulence, cell invasion, and immune evasion, making them potential targets for neglected tropical diseases (NTDs). Cruzain, for instance, is one of the key enzymes involved in vital processes for *T. cruzi* replication and host persistence, positioning its inhibition as a promising therapeutic strategy against Chagas disease [14, 15, 20].

Affecting over 6 million people across 21 Latin American countries, Chagas disease demands innovative approaches. Since drug efficacy varies depending on the parasite's life stage, cruzain has emerged as a prime target, with enzyme inhibitors representing potential drug candidates [13, 14, 15]. Given papain's structural and functional significance, this enzyme could serve as a model for studying related proteases.

In this study, the fungi *F. guttiforme* and *Phytophthora palmivora* were investigated under both axenic and co‐culture conditions, using four distinct culture media: PDB, Czapek, rice, and ISP2. Extracts obtained from these cultures were evaluated for their ability to inhibit the enzyme papain and for antifungal activity against *Colletotrichum horii*. The most promising extracts were selected based on their chemical profiles, analyzed by proton nuclear magnetic resonance (^1^H NMR), and their observed biological activity.

From the most active extracts, twelve chemical compounds were isolated. Among these, the iron complex of fusaric acid (1), the magnesium complex of fusaric acid (2), the magnesium complex of 9,10‐dehydrofusaric acid (**3**), haematocin (**4**), and fusarinolic acid (**7**) were of particular interest. These compounds, due to their unique structural features and bioactivities, were subjected to further biological evaluation, including antifungal assays against *Candida* spp., antiparasitic tests, and inhibition assays targeting the enzyme papain.

## 2 Results and Discussion

### 2.1 Chemical Profile of the Extracts

The chemical profile of the extracts obtained from axenic cultures and co‐cultures in PDB medium was analyzed by ^1^H NMR spectroscopy (Figure S1). The spectra revealed low metabolic diversity, as indicated by the limited number of signals. A similar pattern was observed in the Czapek medium (Figure S2), suggesting that under these conditions, the fungi did not produce compounds of interest.

In contrast, the rice medium exhibited the highest metabolic diversity among the evaluated culture media. The ^1^H NMR spectra (Figure S3) displayed a wide range of signals. The fungus *P. palmivora* (C) predominantly displayed signals corresponding to triglycerides derived from the rice medium itself, as confirmed by comparison with the reference spectrum (blank D). Only low‐intensity signals were observed as metabolites of this fungus. On the other hand, the axenic culture of *F. guttiforme* (A) and the co‐culture (B) showed a greater diversity of signals, suggesting increased metabolite production in these conditions.

In these spectra, aromatic hydrogens were identified in the range of *δ* 6.0–7.2, hydrogens from various amino acids between *δ* 3.5–4.5, and signals in the pyridine core region (*δ* 7.5–8.5). The latter suggests the presence of fusaric acid or its derivatives, compounds commonly produced by fungi of the genus *Fusarium*. In the co‐culture, the relative intensification of these signals, compared to other media, indicates enhanced expression of these metabolites.

The ISP2 medium exhibited the second‐highest diversity of signals in the ^1^H NMR spectra (Figure S4). Relevant patterns were detected for *F. guttiforme* (A) and the co‐culture (B), which exhibited similarities. Signals such as *δ* 5.11, 5.57, 6.00, and 6.08, among others, stood out due to their distinction from the other media, indicating different classes of compounds than those previously observed.

These results underscore the importance of selecting the appropriate culture medium for metabolite production. The diversity of signals obtained demonstrates that the composition of the medium can result in entirely distinct chemical profiles [4, 9].

Another relevant observation is that *P. palmivora* did not produce significant amounts of compounds of interest, as indicated by its 1H NMR spectra, which showed low diversity and high similarity to the blank medium spectrum. In the co‐culture, a metabolic convergence toward *F. guttiforme* metabolites was observed, evidenced by the similarity between the co‐culture and axenic *F. guttiforme* spectra. No signals corresponding to compounds produced by *P. palmivora* were detected in the co‐culture, suggesting that *F. guttiforme* inhibited the growth of *P. palmivora*.

Additionally, stimulation in the production of certain compounds by *F. guttiforme*, in the presence of another microorganism, was observed, as evidenced by the relative intensification of some signals in the co‐culture spectra in rice medium, as in *δ* 7.93, *δ* 8.09, and 8.59 (Figure S3B) compared to the axenic culture (A).

### 2.2 Biological Activity of Extracts and Selection

Antifungal and papain inhibitory activities were evaluated for all twelve extracts to assess the chemical profiles of the extracts and conduct a biological prospection aimed at selecting those with the highest potential for producing compounds of interest. The goal was to identify the most promising extracts for isolation. Papain was selected as the model cysteine protease for enzymatic assays due to its low cost and commercial availability. Antifungal activity assays were performed against *C. horii*, a fungal pathogen responsible for papaya anthracnose, which causes significant post‐harvest losses. The antifungal activity (Table 1) revealed that the co‐culture extracts exhibited equal or greater activity than the axenic cultures of the fungi. This result suggests that competition for nutrients and coexistence in the co‐culture stimulated the production of more bioactive extracts, particularly in terms of antifungal activity. Among the evaluated extracts, those obtained from the ISP2 medium, particularly CoI (co‐culture in ISP2) and F1I (*F. guttiforme* in ISP2), stood out, showing 74.9% and 69.4% inhibition, respectively. The papain inhibitory activity, evaluated at concentrations of 500, 250, and 125 µg/mL (Table 1), demonstrated that the extracts obtained from the ISP2 and rice media, both in the axenic culture of *F. guttiforme* and in the co‐culture, exhibited higher activity. Notably, the extracts of *F. guttiforme* cultivated in rice and ISP2, as well as the co‐culture in ISP2, showed inhibitions of more than 50% at a concentration of 500 µM. The extract of *F. guttiforme* in the ISP2 medium stood out even further, achieving 62.7% inhibition.

**TABLE 1:** Antifungal and papain inhibitory activities of crude extracts.

Code	Papain inhibitory activity (%)			Antifungal activity against *C. horii* (%)
	500 µg/mL	250 µg/mL	125 µg/mL	0.5 mg/mL
F1P	16.2 ± 1, 0	12.1 ± 2, 1	6.5 ± 0, 6	49.3 ± 1, 3
F5P	19.3 ± 4, 5	7.1 ± 1, 8	24.0 ± 2, 5	38.5 ± 0, 4
CoP	31.8 ± 2, 6	27.9 ± 2, 2	17.4 ± 0, 7	53.1 ± 0, 6
F1C	33.5 ± 0, 8	25.9 ± 1, 6	12.1 ± 1, 4	49.9 ± 0, 3
F5C	32.4 ± 1, 5	30.4 ± 4, 4	18.5 ± 1, 7	40.0 ± 1, 3
CoC	34.1 ± 1, 2	30.2 ± 1, 9	16.8 ± 4, 4	55.1 ± 0, 8
F1A	54.1 ± 2, 1	40.1 ± 3, 6	10.6 ± 2, 5	46.7 ± 1, 3
F5A	12.4 ± 3, 2	20.0 ± 1, 2	21.7 ± 1, 5	34.0 ± 1, 1
CoA	47.2 ± 0, 8	28.5 ± 0, 7	11.5 ± 1, 5	37.2 ± 0, 7
F1I	62.7 ± 2, 0	48.2 ± 2, 3	38.8 ± 0, 8	69.4 ± 1, 1
F5I	38.8 ± 2, 1	27.4 ± 0, 7	17.6 ± 1, 0	47.1 ± 2, 8
CoI	53.0 ± 2, 1	44.2 ± 0, 8	31.3 ± 3, 0	74.9 ± 1, 4

Where: F1 = *F. guttiforme*, F5 = *P. palmivora*, Co = coculture, P = PDB medium, C = czapek medium, A = rice medium, and I = ISP2 medium.

Based on the results of the biological activities, the most promising extracts were those derived from the ISP2 and rice media for *F. guttiforme*. Considering both the chemical profiles and biological activities, the following extracts were selected for the fractionation process: CoA and F1I

### 2.3 Isolation and Identification of Compounds

**FIGURE 1:**
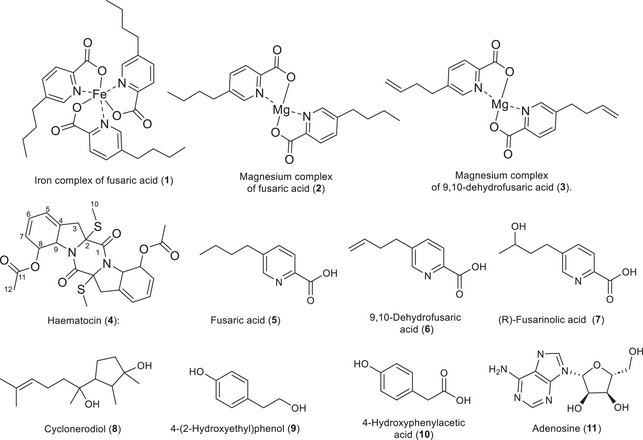
Structure of the isolated compounds.

The isolation of the extract from the ISP2 medium led to compounds unique to this medium, such as fusaric acid and its derivatives, as well as some compounds similar to those found in the rice medium (Figure 1).

Compound **1** was isolated as a brown solid and exhibited a ^1^H NMR spectrum similar to fusaric acid, displaying all signals of the former. However, the signals of the pyridine core showed different chemical shifts. These signals are δ_H5_ 8.50 (1H, s), δ_H3_ 8.11 (1H, d, *J* = 8.0 Hz), and δ_H4_ 7.91 (1H, dd, *J* = 8.0 and 1.8 Hz), with higher chemical shifts indicating more deshielded hydrogens compared to fusaric acid and its magnesium complex (Figure S5) [9]. Signals of the n‐butyl group were observed at δ_H7_ 2.75 (2H, m), δ_H8_ 1.65 (2H, m), δ_H9_ 1.39 (2H, m), and δ_H10_ 0.96 (3H, t, *J* = 7.4 Hz), indicating that the n‐butyl group remains intact in the compound. Given these observations, there was a possibility that this compound was another metal‐complexed derivative. High‐resolution mass spectrometry (HRMS) (Figure S6) confirmed the presence of three units of fusaric acid complexed with iron with the ion *m/z* 613.1842, [M + Na]⁺, corresponding to the molecular formula C_30_H_36_FeN_3_O_6_. Thus, the compound was identified as an iron complex of fusaric acid, reported for the first time as a natural product. In the literature, a study on the complexation of fusaric acid with different metals showed that compound **1** exhibits a MIC of 3.4 × 10^−2^ against *Mycobacterium bovis* [21].

Two additional compounds were also isolated but are not detailed in this work, as we had previously described them in a publication by Mazucato and Vieira: the magnesium complex of fusaric acid (2) and the magnesium complex of 9,10‐dehydrofusaric acid (**3**). These compounds were also isolated from the rice medium in the axenic culture extract. For the first time, they are reported in the rice and ISP2 media as products of the fungus *F. guttiforme*. From the crude extract of the axenic culture of *F. guttiforme* in ISP2 medium, uracil was isolated. As this is a common compound, its spectrum was not shown. Additionally, a compound named haematocin (**4**) was isolated, which stands out as a strong candidate for papain inhibition in the extracts.

The ^1^H NMR spectrum of compound **4** (Figure S7) displayed three signals corresponding to olefinic hydrogens: δ_H5_ 6.00 (1H, m), δ_H6_ 6.02 (1H, m), and δ_H7_ 5.57 (1H, d, *J* = 9.0 Hz). Two signals of hydrogens attached to carbons with heteroatoms were identified: δ_H8_ 6.08 (1H, d, *J* = 14.4 Hz, oxygenated) and δ_H9_ 5.11 (1H, d, *J* = 14.4 Hz, nitrogenated). Furthermore, two methyl signals were observed, one alpha to a carbonyl group (δH_12_ 2.03, 3H, s) and the other bonded to sulfur (δ_H10_ 2.23, 3H, s). Methylene hydrogens were detected at δ_H3_ 2.98 (2H, m), correlated to the corresponding carbon (δ_C_ 40.56), as confirmed by HSQC (Figure S9).

**FIGURE 2:**
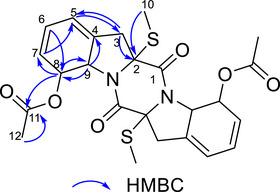
Heteronuclear multiple bond coherence (HMBC) correlations for compound **4**.

Through HSQC correlations, all hydrogens were assigned to their respective carbons, as shown in Table 2. Heteronuclear multiple bond coherence (HMBC) correlations (Figure S9) enabled the assignment of the remaining carbon chemical shifts. All observed correlations are presented in Table 2 and Figure 2. Key structural correlations include H_10_ (*δ*_H_ 2.23) with C‐2 (*δ*_C_ 75.39) and H_3_ (*δ*_H_ 2.98) with C‐2 (*δ*_C_ 75.39), C‐4 (*δ*_C_ 136.60), and C‐5 (*δ*_C_ 120.00). Additionally, H_9_ (*δ*_H_ 5.11) showed correlations with C‐4 (*δ*_C_ 136.60) and C‐8 (*δ*_C_ 76.06), while H_8_ (*δ*_H_ 6.08) correlated with C‐7 (*δ*_C_ 128.54), C‐9 (*δ*_C_ 65.18), and C‐11 (*δ*_C_ 170.60), connecting the structure.

**TABLE 2:** Data Obtained for Compound **4**.

*N*_position_	^13^C/^1^H—*δ*_H_ (integral, multiplicity, *J* = Hz)	HMBC Correlations	^1^ ^3^C/^1^H—*δ*_H_ (integral, multiplicity, *J* = Hz) [22]
1	165.86	—	164.90
2	75.39	—	74.14
3a	40.56/2.98 (2H, m)	18.00; 75.39; 120.00; 136.60	40.13/2.8 (1H, ld, *J* = 16.1)
3b			3.01 (1H, dd, *J* = 16.1 e 1.2)
4	136.60	—	133.98
5	120.00/6.00 (1H, m)	56.00; 65.18; 128.54	119.98/5.94 (1H, m)
6	126.34/6.02 (1H, m)	76.16; 136.60	125.11/5.96 (1H, m)
7	128.54/5.57 (1H, dl *J* = 9.5)	65.18; 120.00	128.04/5.58 (1H, ld, *J* = 9.2)
8	76.16/6.08 (1H, dl, *J* = 14.4)	65.18; 126.34; 128.54; 170.00	75.39/6.13 (1H, ld, *J* = 14.8)
9	65,18/5.11 (1H, dl, *J* = 14,4)	76.16; 136.60	64.25/5.16 (1H, ld, *J* = 14.8)
10	14.48/2.23 (3H, s)	75.39	14.34/2.25 (3H, s)
11	170.6	—	170.43
12	21.41/2.03 (3H, s)	170.60	21.34/2.11 (3H, s)
**Freq/Solvent**	**Acetone–d_6_ 600 MHz**		**400 MHz/CDCl_3_**

HRMS data (Figure S9) confirmed the compound's symmetry. Comparison with literature information [22], listed in Table 2, allowed the identification of compound **4** as haematocin.

During the fractionation of the ISP2 medium, a ^1^H NMR spectrum of a compound with signals similar to haematocin was observed. However, haematocin exhibits a plane of symmetry, resulting in half the number of hydrogen signals in the ^1^H NMR spectrum, which was not observed for this new compound. By comparing the integrals of the hydrogen signals, the absence of an ester group in haematocin was noted, suggesting an asymmetric molecule related to cladosporin. However, it was not possible to achieve satisfactory purity for this compound.

Compounds **5**, **6**, and **8** were obtained from the axenic culture of *F. guttiforme* and the co‐culture in the ISP2 medium. Compounds **5** and **6** have been commonly reported for this fungal species, including in our previous work it was isolated from PDB medium [9]. Compound **8** was observed in the same medium after the use of epigenetic modifiers [9]. Compound **8** was identified as cyclonerodiol based on comparison of its ^1^H NMR spectrum with those from the literature [23], a compound commonly found in fungi. Therefore, its spectra were not detailed. Compound **8** did not exhibit significant biological activity, showing only weak antifungal activity against *Cryphonectria parasitica* [24].

Compound **7**, was isolated from the rice medium, exhibited characteristic signals in the ^1^H NMR spectrum (Figure S10) of a disubstituted pyridine core: δ_H3_ 7.92 (1H, s), δ_H4_ 7.72 (1H, s), and δ_H5_ 8.43 (1H, s), consistent with fusaric acid or its derivatives isolated from *F. guttiforme*. Additionally, the compound showed characteristic signals of an n‐butyl group: *δ*_H7_ 2.70 (1H, m) and 2.80 (1H, m); δ_H8_ 1.70 (2H, m); δ_H10_ 1.17 (3H, d, *J* = 6.0 Hz). The signal at *δ*_H_ 3.70 (1H, m) indicates the presence of a hydroxyl group at position H9, evidenced by the doublet multiplicity at δ_H10_ 1.17. Comparison with literature data allowed this compound to be identified as (R)‐fusarinolic acid (${\left[\alpha \right]}_{D}^{27}$–25.8) [25]. This compound has been previously tested for antibacterial activity [26] and plant‐parasitic nematode activity [27], but no significant activity was observed.

Other compounds were also isolated from the co‐culture extract in rice medium: 4‐(2‐hydroxyethyl)phenol (**9**), 4‐hydroxyphenylacetic acid (**10**), and adenosine (**11**) (Figure 1). However, as these compounds are considered simple and widely described in the literature, their data were not presented. Additionally, amino acids and cyclodipeptides such as cyclo(L‐Val‐L‐Pro), cyclo(L‐Leu‐L‐Pro), and cyclo(L‐Leu‐L‐Pro) diketopiperazine were isolated, which had previously been reported by our group and showed papain inhibition of 10%, 94%, and 3% at a concentration of 50 µM, respectively [28].

Table 3 correlates compounds obtained and the medium in which they were isolated. From it, variations in the presence and quantity of compounds between the different media can be observed. Notably, there are compounds exclusive to each medium, such as the Iron complex of fusaric acid (**1**) and haematocine (**4**), which were obtained only in the ISP2 medium. On the other hand, compounds such as cyclonerodiol (**8**), 4‐(2‐hydroxyethyl)phenol (**9**), fusaric acid (**5**), and its derivatives were observed in both media, but in different quantities.

**TABLE 3:** Comparison of compounds obtained in each extract.

Medium	Compound														
	1	2	3	4	5	6	7	8	9	10	11	12	13	14	Others not identified.
ISP2	x	x	x	x	x	x		x	x			x	x	x	x
Rice		x	x		x		x	x	x	x	x	x	x		x

Additionally, diketopiperazines were isolated in both media. Compounds such as Cyclo(Pro‐Val) and Cyclo(Pro‐Leu) had previously been isolated by our group in Czapek medium [9], and now another diketopiperazine, Cyclo(Val‐Phe), was isolated in the ISP2 medium. Other amino acids and peptides were also detected in other fractions, but were not conclusively isolated or identified. Even if not fully characterized, the presence of these compounds indicates the complexity of the secondary metabolism of the analyzed microorganisms and opens avenues for future studies to elucidate their structures and biological activities.

### 2.4 Biological Activity of the Compounds

The compounds were tested according to their availability and the mass obtained. For some compounds, a larger quantity was required for elucidation and identification, which prevented the evaluation of all planned activities.

### 2.5 Antifungal Activity

Compounds **1–5, 7–11** were evaluated for antifungal activity against three species of *Candida*. Overall, low biological activity was observed, with only the fusaric acid derivatives and their metal demonstrating inhibitory effect at the tested concentrations against *C. albicans*. Fusaric acid (**4**) and its iron complex of fusaric acid (**1**) showed a minimum inhibitory concentration (MIC) of 100 µg/mL, while the magnesium complex of fusaric acid (**2**) and the magnesium complex of 9,10‐dehydrofusaric acid (**3**) exhibited MIC values of 50 µg/mL. The remaining compounds did not exhibit any activity against *C. auris*, *C. parapsilosis*, or *C. albicans* at the maximum tested concentration of 200 µg/mL. Fluconazole was used as the reference antifungal control.

### 2.6 Papain Inhibitory Activity

Compounds **1**, **2**, **4**, and **7–11** were tested for their papain inhibitory activity, and the results are summarized in Table 4. Compounds **1** and **2** showed a strong inhibitory effect, with 88.6% and 94.0% inhibition, respectively. These values are significantly higher than the previously reported inhibition for fusaric acid (58% at 200 µM) [17]. Other compounds with significant activity included **4** and **8**, with 76.6% and 89.0% inhibition, respectively. Given their abundance in the most active extracts, these compounds may contribute to the observed antifungal activity. The IC_50_ values for the most active compounds are shown in Table 5. The IC_50_ values further support the efficacy of these compounds. Compound **1** exhibited the strongest inhibition, with an IC_50_ value of 14.1 µM, followed by compound **2** (17.5 µM) and compound **8** (25.9 µM). Although compound **4** exhibited a higher IC_50_ value (47.7 µM), it still demonstrated notable inhibitory activity compared to the other tested compounds.

**TABLE 4:** Inhibitory activity of papain by the compounds.

Compound	% inhibition (100 µM)	IC_50_ (µM)
1	88.6 ± 4,0	14.1 ± 1,9
2	94 ± 7,9	17.5 ± 1,0
4	76.6 ± 1,0	47.7 ± 1,5
7	17.2 ± 3,6	—
8	89,0 ± 2,7	25.9 ± 1,3
9	28.4 ± 11,4	—
10	0.7 ± 0,7	—
11	7.3 ± 3,4	—
E‐64	—	7,2 ± 1,1 (nM)

**TABLE 5:** Anti‐*Trypanosoma cruzi* activity of the compounds.

Compound	IC_50_ (µM)	CC_50_ (µM)	SI
2	12.19 ± 0.4	> 64	> 5.25
4	13.32 ± 1.0	> 64	> 4.80
5	20.86 ± 0.3	> 64	> 3.06
7	60.07 ± 0.6	> 64	> 1.06
8	22.74 ± 1.1	> 64	> 2.81
Benznidazole	2.24 ± 0.9	—	—

### 2.7 Anti‐*T. cruzi* Activity

Among the tested compounds (Table 5), compound **2** showed promising activity, with an IC_50_ of 12.19 µM, which is superior to that of compound 5 (20.86 µM). This suggests that complexation may positively influence anti‐*T. cruzi* activity. Compound **7**, on the other hand, exhibited lower activity (IC_50_ of 60.07 µM), which may be related to the hydroxylation in its structure. Notably, compound **4** was one of the most promising, with an IC_50_ of 13.32 µM, marking the first report of its activity against a protozoan. This justifies further studies to evaluate its efficacy against other neglected tropical diseases. Compound **8** also showed activity, reported for the first time against a protozoan, with an IC_50_ of 22.74 µM, and it could be tested for other diseases.

Compound **7** exhibited lower antifungal and anti‐*T. cruzi* activities compared to fusaric acid. These results suggest that its reduced efficacy may be related to detoxification processes, as hydroxyl groups' presence tends to reduce compounds' toxicity, as previously reported [9, 29, 30, 31].

The compounds were evaluated for cytotoxicity in human fibroblasts (HFF‐1) at the maximum tested concentration of 64 µM. None exhibited significant toxicity in this range (CC_50_ > 64 µM), indicating a safe profile in mammalian cells. The selectivity index (SI) revealed notable differences among the compounds. Compound **7** displayed the lowest selectivity (SI > 1.06), consistent with its poor antiparasitic efficacy relative to its residual cytotoxicity. Compounds **5** and **8** exhibited moderate selectivity indices (SIs > 3), whereas compounds **2** and **4** displayed the highest SI values among the tested compounds (SI > 4.80–5.25).

## 3 Conclusions

This study demonstrates the effectiveness of cultivating *F. guttiforme* and *P. palmivora* under diverse media and co‐culture conditions to enhance the production of bioactive secondary metabolites. The isolation of compounds such as the fusaric acid complexes with iron (**1**) and magnesium (**2**), haematocin (**5**), fusarinolic acid (**7**), and cyclonerodiol (**8**) revealed significant papain inhibitory and anti‐*T. cruzi* activities, with compounds **2** and **4** showing particularly promising IC_50_ values. Additionally, antifungal activity against *Candida* spp. was observed, especially for the magnesium complex of fusaric acid (**2**) and the complex of 9,10‐dehydrofusaric acid (**3**), both of which exhibited MICs of 50 µg/mL. These findings underscore the potential of fungal co‐cultivation and culture medium optimization as strategies for discovering novel protease inhibitors and antiparasitic agents. The findings of this study support the development of novel therapeutic candidates targeting neglected tropical diseases and fungal infections.

## 4 Experimental

### 4.1 Fungal Strains

The fungal strains *Fusarium guttiforme* (MMBF 03/07) and *Phytophthora palmivora* (MMBF 13/79) were obtained from the microbiological collection of the Biological Institute of São Paulo, Brazil. Both strains were subsequently maintained in our laboratory's culture collection under standard preservation conditions.

### 4.2 Cultivation of *F. guttiforme* and *P. palmivora* in Axenic and Co‐culture Systems

The axenic and co‐culture experiments were conducted using four distinct media: PDB (Potato Dextrose Broth, Himedia), Czapek, rice, and ISP2. The Czapek medium was prepared according to the following composition per liter: 2.0 g sodium nitrate, 0.5 g magnesium sulfate, 0.5 g potassium chloride, 0.01 g ferrous sulfate, 1.0 g dipotassium phosphate, 30.0 g sucrose, and 20.0 g yeast extract (Himedia), with the final pH adjusted to 5.0. The rice medium was prepared by combining 90.0 g of rice with 90 mL of distilled water. The ISP2 medium was formulated with 4.0 g glucose (Synth), 10.0 g malt extract (Acumedia), and 4.0 g yeast extract (Himedia) per liter.

For initial fungal propagation, Potato Dextrose Agar (PDA) plates were prepared, sterilized by autoclaving at 125°C for 20 min, and poured into sterile 10 cm Petri dishes. Each fungal strain was inoculated onto the center of the PDA plates and incubated at 25°C ± 1°C, in the absence of light, for 7 days. After the incubation period, six mycelial agar discs (0.5 cm diameter) were aseptically excised from the actively growing edges of the colonies using a sterile transfer tube. These discs were then inoculated into 500 mL Erlenmeyer flasks containing 200 mL of the respective liquid or solid medium.

For each medium, six Erlenmeyer flasks were used for axenic cultures of *F. guttiforme* and *P. palmivora*, as well as for their co‐culture. Two additional flasks without fungal inoculation were prepared as controls for each medium, bringing the total to twenty flasks per medium. All cultures were incubated at 25°C ± 1°C, under static conditions and in the absence of light. The incubation period was 28 days for liquid media and 21 days for the rice medium. For the ISP2 medium, large‐scale cultivation of *F. guttiforme* was performed using 60 Erlenmeyer flasks under identical conditions to ensure sufficient biomass and metabolite production for subsequent analyses.

### 4.3 Filtration, Extraction, and Crude Extract Preparation

After the designated cultivation period, the cultures were vacuum‐filtered using a vacuum pump and quantitative filter paper. The mycelium was discarded in appropriate biological waste bags, and liquid‐liquid partitioning of the broth was performed using 300 mL of ethyl acetate for every 600 mL of aqueous phase, repeated three times, yielding 900 mL of organic phase. The organic phase was then washed three times with 450 mL of water. For the rice medium, 150 mL of ethyl acetate was added to the cultivation flask, followed by maceration with a glass rod to ensure greater contact area. The mixture was sonicated for 5 min and filtered through quantitative filter paper. This process was repeated three times, yielding 450 mL of organic phase. The solvent was evaporated using a rotary evaporator equipped with a vacuum pump to obtain the crude extract. For the large‐scale ISP2 medium cultivation, the same extraction steps were followed.

### 4.4 Chemical Profile Evaluation of Crude Extra

^1^H NMR analyses were performed using a Bruker Advance spectrometer (400 MHz) at the Departamento de Química from Faculdade de Filosofia, Ciências e Letras de Ribeirão Preto (FFCLRP‐USP). Deuterated acetone‐d₆ (Merck/Aldrich) was used as the solvent to evaluate the chemical profile of the crude extracts.

For the characterization of isolated compounds, one‐dimensional (^1^H) and two‐dimensional (HMBC and HSQC) nuclear magnetic resonance (NMR) analyses were conducted using a Bruker DRX500 spectrometer operating at 500 MHz. Samples were prepared using deuterated solvents, including acetone–d_6_, CDCl_3,_ and methanol–d_4_ (Merck/Aldrich).

Additionally, high‐resolution mass spectrometry (HRMS) was performed using a Bruker microTOF Q II spectrometer equipped with electrospray ionization (ESI) and a time‐of‐flight (TOF) analyzer. This equipment is located at the Laboratório de Química Orgânica of Faculdade de Ciências Farmacêuticas de Ribeirão Preto.

### 4.5 Evaluation of the Biological Activity of the Crude Extracts

#### 4.5.1 Antifungal Activity Screening

Antifungal activity was assessed using the agar dilution method, adapted from previously described protocols to fit the experimental conditions in this study [32, 33]. Sterile Petri dishes containing PDA medium were prepared by incorporating the fungal extracts to achieve a final concentration of 0.5 mg/mL. Mycelial plugs were aseptically transferred onto the surface of plates and incubated at 28°C for 7 days. A control plate containing PDA without extract was included for comparison. All procedures were performed in triplicate. Fungal growth inhibition was calculated by measuring the average colony diameter and comparing it to the control. The percentage of inhibition was calculated using the following formula:

$$\begin{array}{c} \mathrm{Inhibition}(\%)=(\mathrm{Dc}-\mathrm{Dt})/\mathrm{Dc})\times 100, \end{array}$$

where Dc is the average colony diameter of the control group, and Dt is the average colony diameter in the presence of the test extract.

#### 4.5.2 Enzymatic Inhibition Assay

The inhibitory activity against the enzyme papain was evaluated following the validated method in the literature [34]. This method involves the proteolytic cleavage of casein present in powdered milk, uniformly incorporated into agar‐nutrient plates. Wells were created in the plates and divided into distinct groups. The negative control group consisted of papain as the protease, which was prepared in sodium phosphate buffer and dimethyl sulfoxide (DMSO). The positive control group included papain and a standard irreversible protease inhibitor, E‐64. The experimental groups consisted of fungal extracts at concentrations of 500, 250, and 125 µg/mL, compared against the standard inhibitor.

The enzymatic solution was added to the wells, and a 24‐h incubation period was allowed for enzyme diffusion. During this time, casein cleavage by papain occurred. Subsequently, diluted HCl was added to denature the remaining casein, resulting in the formation of a clear halo around the wells. The relationship between enzyme activity and the diameter of the inhibition halo was established. The percentage of proteolytic inhibition was calculated as follows:

$$\begin{array}{c} \%\mathrm{IE}=100(1\text{--}A_{\mathrm{extract}}/A_{\mathrm{enzyme}}), \end{array}$$

where A_extract_ is the area of the clear halo for the extract, and A_enzyme_ refers to papain with DMSO without the action of an inhibitor.

#### 4.5.3 Isolation of Compounds **1**–**11**

The crude extracts from the co‐culture in rice medium (3.5 g) and the axenic culture of *F. guttiforme* in ISP2 medium (40 mg—small scale and 500 mg—large scale) were fractionated by vacuum liquid chromatography (VLC). Glass Büchner funnels with porous plates (500 mL, 10 cm diameter, 24 cm height; and 250 mL, 7.5 cm diameter, 18.5 cm height) were used with silica gel (40–70 mesh) as the stationary phase. Elution was performed using gradients of 300 mL of organic solvents, hexane, and ethyl acetate as the mobile phase. The gradient started with 100% hexane (VLC1), followed by 66:33 hexane:ethyl acetate (VLC2), 33:66 hexane:ethyl acetate (VLC3), 100% ethyl acetate (VLC4), and finally 400 mL of methanol (VLC5). For the axenic culture in ISP2 medium, the same elution system was used, but with half of the solvent volume.

The VLC4 fractions from both extracts were further separated by size‐exclusion chromatography using Sephadex LH‐20 (Amersham Pharmacia Biotech AB) as the stationary phase in a glass column (2.5 cm diameter, 60 cm height). The Sephadex was suspended in methanol (analytical grade) and packed to occupy 53 cm of the column height. Fractions were collected every 5 mL until completion. Subsequently, the samples were purified by High‐performance liquid chromatography with diode array detection (HPLC‐DAD). A Shimadzu LC‐6AD pump and a semi‐preparative C‐18 column (25 cm × 10 mm, 5 µm—Phenomenex Luna C‐18 100A) were used with a flow rate of 4.0 mL/min. The elution system employed a gradient of water and methanol, starting with 30% MeOH, reaching 100% MeOH in 25 min, and maintained for 5 min, totaling 30 min. From the VLC4 fractions of the axenic culture of *F. guttiforme* in ISP2 medium, compounds **4** (29.0 mg), **8** (6.6 mg), and **9** (5.8 mg) were obtained. From the co‐culture extract in rice medium, compounds **8** (4.3 mg), **9** (20.2 mg), **10** (13.4 mg), and **11** (15.8 mg) were isolated.

The VLC5 fractions from both extracts were also subjected to the same size‐exclusion chromatography above and purified by HPLC‐DAD under identical conditions, except for the mobile phase, which started with 5% MeOH and reached 100% MeOH in 25 min, maintained for 5 min, totaling 30 min. From the VLC5 fractions of the axenic culture of *F. guttiforme* in ISP2 medium, compounds **1** (7.2 mg), **2** (3.2 mg), **3** (2.2 mg), **5** (10.8 mg), and **6** (5.9 mg) were obtained. From the co‐culture extract in rice medium, compounds **2** (17.0 mg), **3** (4.2 mg), **5** (3.7 mg), and **7** (5.7 mg) were isolated.

Iron complex of fusaric acid (**1**): brown solid, molecular formula C_30_H_36_FeN_3_O_6_. ^1^H NMR (400 MHz, acetone–d₆): δ_H3_ 8.11 (1H, d, *J* = 8.0 Hz), δ_H4_ 7.91 (1H, dd, *J* = 8.0 and 1.8 Hz), δ_H5_ 8.50 (1H, s), δ_H7_ 2.75 (2H, m), δ_H8_ 1.65 (2H, m), δ_H9_ 1.39 (2H, m), δ_H10_ 0.96 (3H, t, *J* = 7.4 Hz). HRESIMS: *m/z* 613.1842 [M + Na]⁺ (error +1.5 ppm, calcd for C_24_H_26_FeN_2_O₆Na^+^, 613.1851).

Haematocin (**4**): white amorphous powder, molecular formula C_24_H_26_N_2_O,_6_S_2_. ^1^H NMR data are summarized in Table 2. HRESIMS: *m/z* 525.1122 [M + Na]⁺ (error –1.5 ppm, calcd for C_24_H_26_N_2_O₆S_2_Na^+^, 525.1130).

Fusarinolic Acid (**7**): amorphous solid, molecular formula C_10_H_13_NO_3_, ${\left[\alpha \right]}_{D}^{27}$ –25.8 (c 1.00, MeOH) [lit. ${\left[\alpha \right]}_{D}^{27}$ +19.5° (c 1.5, MeOH) [25]]. ^1^H NMR (400 MHz, acetone–d₆): δ_H3_ 7.92 (1H, s), δ_H4_ 7.72 (1H, s), δ_H5_ 8.43 (1H, s), δ_H7_ 2.70 (1H, m) and 2.80 (1H, m), δ_H8_ 1.70 (2H, m), δ_H9_ 3.70 (1H, m), δ_H10_ 1.17 (3H, d, *J* = 6.0 Hz).

### 4.6 Evaluation of the Biological Activity of Isolated Compounds

#### 4.6.1 Antifungal Activity

The antifungal activity of compounds **1–5, 7–11** was evaluated using the broth microdilution method in 96‐well plates, following the Clinical Laboratory Standard Institute (CLSI) guidelines M27A3 [35, 36], with minor modifications. The assay was performed against *Candida auris* (CDC B11903), *Candida parapsilosis* (ATCC 22019), and *Candida albicans* (ATCC 64548), and based on the determination of the minimum inhibitory concentration (MIC).

Each compound was initially dissolved in DMSO at a stock concentration 100 times higher than the highest concentration tested. Working solutions were subsequently prepared in RPMI 1640 culture medium (pH 7.0), supplemented with L‐glutamine, devoid of bicarbonate, and containing phenol red as a pH indicator, to yield a 2 × concentration. Serial two‐fold dilutions were made to obtain final concentrations ranging from 3.125 to 200.00 µg/mL. Fluconazole was used as a reference antifungal control.

Aliquots of 100 µL of each 2 × test solution were dispensed into the wells of the microdilution plates. Fungal suspensions were prepared in autoclaved distilled water and adjusted to a final concentration of 5.0 × 10^3^ conidia or blastoconidia/mL. After inoculation, plates were incubated at 37°C. Fungal growth was visually assessed after 24 and 48 h. The MIC was defined as the lowest concentration of the compound that completely inhibited visible fungal growth. All assays were performed in duplicate.

#### 4.6.2 Papain Inhibitory Activity

Compounds **1**, **2**, **4**, and **7–11** were tested against papain inhibitory activity. Papain served as the model cysteine protease in the protease inhibition assay. Enzyme activity was assessed via real‐time fluorometric monitoring of 7‐amino‐4‐methylcoumarin (MCA) release during substrate cleavage [37]. Hydrolysis of the fluorogenic substrate Z‐Phe‐Arg‐MCA (ZFR‐MCA) was tracked in black 96‐well plates using a spectrofluorometer (λex/λem = 380/460 nm) (SpectraMax M3, Molecular Devices).

Each reaction well contained 5 µL papain (80 nM), 2 µL dithiothreitol (DTT, 500 mM), and 158 µL sodium acetate buffer (100 mM, pH 5.5, 5 mM EDTA). After a 5‐minute pre‐incubation at 27°C, 5 µL of test compound (dissolved in DMSO) or DMSO (negative control) was added. In the initial screening, all compounds were tested at a fixed concentration of 100 µM to evaluate their inhibitory potential. Following a second 5‐minute incubation at 27°C, 30 µL ZFR‐MCA (0.6 mM) was added, and fluorescence was recorded every 300 s using a SpectraMax M3 microplate reader (Molecular Devices). This concentration guided subsequent serial dilutions to determine the IC_50_ (half‐maximal inhibitory concentration).

Compounds exhibiting over 70% inhibition of papain activity were selected for further analysis to determine their half‐maximal inhibitory concentration (IC_50_). For this, a serial dilution series was prepared from the active compounds, and the assay was conducted as previously described. Final assay conditions (200 µL/well) yielded effective compound concentrations of 100 µM to 1.56 µM for IC_50_ determination. Papain's molar concentration was validated via titration with the irreversible inhibitor E‐64 (positive control) from 1.0 µM to 31.25 nM. All experiments included triplicate measurements across two independent trials (total *n* = 6).

#### 4.6.3 Anti‐*T. cruzi* Activity

Compounds **1**, **2**, **4**, **7**, and **9** were subsequently tested for their anti‐*T. cruzi* activity [38]. The evaluation utilized the genetically modified *T. cruzi* Tulahuen lacZ strain, engineered to express the *lacZ* gene encoding β‐galactosidase. Viable intracellular parasites express this enzyme, which hydrolyzes the substrate chlorophenol red‐β‐D‐galactopyranoside (CPRG) to release chlorophenol red, a chromogenic product quantified spectrophotometrically at 570 nm. The absorbance intensity directly correlates with the number of metabolically active parasites present during the assay.

Axenic cultures of *T. cruzi* epimastigotes (strain MHOM/CH/00/Tulahuen C2, *lacZ*) were grown in LIT medium containing 10% fetal calf serum (FCS) at 28°C. During the exponential growth phase, parasites were collected via centrifugation (250 × *g*, 10 min), resuspended in Grace's insect medium supplemented with 10% FCS, and differentiated into metacyclic trypomastigotes over 5–14 days. Differentiated parasites were harvested and used to infect Human Foreskin Fibroblasts (HFF‐1), cultured in Dulbecco's Modified Eagle Medium (DMEM) with 10% FCS under 5% CO_2_ at 37°C.

For infection assays, HFF‐1 cells were plated in 96‐well plates (5 × 10⁴ cells/well) in 80 µL phenol red‐free DMEM and incubated overnight. Metacyclic trypomastigotes (5 × 10⁵ parasites/well in 20 µL) were added, followed by 24‐hour incubation. After this period, trypomastigotes differentiate into intracellular amastigotes. Non‐infective parasites were removed by replacing the medium with 100 µL fresh DMEM. Test compounds, at varying concentrations, were then applied to determine IC_50_ values against intracellular amastigotes, with benznidazole as the positive control. After 5 days of incubation (37°C, 5% CO_2_), 50 µL of 1 mM CPRG and 0.1% IGEPAL CA‐630 (a non‐ionic detergent) were added. β‐Galactosidase activity, reflecting parasite viability, induced a colorimetric shift from yellow to red, measured at 570 nm. Growth inhibition percentages were calculated relative to untreated controls. Benznidazole was used as the positive control and DMSO as the negative control.

Cytotoxicity assays: HFF‐1 fibroblasts (provided by Rio de Janeiro Cell Bank (BCRJ)) were seeded at a density of 2 × 10^3^ cells per well in 100 µL of DMEM and incubated overnight at 37°C in a 5% CO_2_ atmosphere. Subsequently, test compounds were added in serial dilutions, followed by a 120‐hour incubation period under the same conditions. Each plate included negative controls and doxorubicin as a positive control. After incubation, 15 µL of MTS reagent (CellTiter96) was added to each well, and plates were incubated for an additional 4 h. Absorbance was measured at 490 nm using a microplate reader. The percentage of growth inhibition was calculated relative to the absorbance of the negative control wells.

## Author Contributions

**Vitor Souza Mazucato**: conceptualization, investigation, methodology, supervision, writing – original draft. **Ludmilla Tonani**: methodology. **Marcia Regina von Zeska Kress**: methodology. **Adriano Defini Andricopulo**: methodology. **Gisele Barbosa**: methodology. **Renata Krogh**: methodology. **Leonardo Luiz Gomes Ferreira**: methodology. **Paulo Cezar Vieira**: conceptualization, methodology, writing – review and editing. All authors have read and agreed to the published version of the manuscript.

## Conflicts of Interest

The authors declare no conflicts of interest.

## Supporting information




**Supporting File1**: cbdv70414‐sup‐0001‐SuppMat.docx.

## Data Availability

The data that support the findings of this study are available in the Supporting Information of this article.
