# Supplementary material for: Co‐cultivation and Medium Optimization: A Strategy for Discovering Fungal‐derived Protease Inhibitors
Source: Chem Biodivers. 2025 Sep 2;22(12):e01135. doi: 10.1002/cbdv.202501135 (PMC12715984; doi:10.1002/cbdv.202501135)
Supplement: Supplementary file 1 — Supporting File1: cbdv70414‐sup‐0001‐SuppMat.docx. [file CBDV-22-e01135-s001.docx]

**Table of contents**

**Figure S1:** ¹H NMR spectra of extracts from the PDB medium after 28 days (acetone-d₆, 400 MHz).

**Figure S2:** ¹H NMR spectra of extracts from the Czapek medium after 28 days (acetone-d₆, 400 MHz).

**Figure S3:** ¹H NMR spectra of extracts from the rice medium after 21 days (acetone-d₆, 400 MHz).

**Figure S4:** ¹H NMR spectra of extracts from the ISP2 medium after 28 days (acetone-d₆, 400 MHz).

**Figure S5.** Comparison of the 1H NMR spectra in methanol-d_4_ at 500 MHz of fusaric acid (**5**) (A), the fusaric acid-magnesium complex **2** (B), and the fusaric acid-iron complex **1** (C).

**Figure S6.** HRESIMS spectrum of compound **1.**

**Figure S7**. ^1^H NMR spectra of compound **4** (Acetone-d_6,_ 500 MHz).

**Figure S8**. HSQC spectra of compound **4** (Acetone-d_6,_ 500 MHz).

**Figure S9**. HMBC spectra of compound **4** (Acetone-d_6,_ 500 MHz).

**Figure S10.** HRESIMS spectrum of compound **4.**

**Figure S11**. ^1^H NMR spectra of compound **7** (Methanol-d_4,_ 500 MHz).

**A**

**B**

**C**

**D**

**Figure S1:** ¹H NMR spectra of extracts from the PDB medium after 28 days (acetone-d₆, 400 MHz).

Where A is Axenic culture of *F. guttiforme*, B is Co-culture extract of *F. guttiforme* and *P. palmivora*, C is Axenic culture of *P. palmivora* and D is Medium blank (absence of fungi).

**Figure S2:** ¹H NMR spectra of extracts from the Czapek medium after 28 days (acetone-d₆, 400 MHz).

**A**

**B**

**C**

**D**

Where A is Axenic culture of *F. guttiforme*, B is Co-culture extract of *F. guttiforme* and *P. palmivora*, C is Axenic culture of *P. palmivora* and D is Medium blank (absence of fungi).

**A**

**B**

**C**

**D**

**Figure S3:** ¹H NMR spectra of extracts from the rice medium after 21 days (acetone-d₆, 400 MHz).

Where A is Axenic culture of *F. guttiforme*, B is Co-culture extract of *F. guttiforme* and *P. palmivora*, C is Axenic culture of *P. palmivora* and D is Medium blank (absence of fungi).

**Figure S4:** ¹H NMR spectra of extracts from the ISP2 medium after 28 days (acetone-d₆, 400 MHz).

**A**

**B**

**C**

**D**

Where A is Axenic culture of *F. guttiforme*, B is Co-culture extract of *F. guttiforme* and *P. palmivora*, C is Axenic culture of *P. palmivora* and D is Medium blank (absence of fungi).

**Figure S5.** Comparison of the 1H NMR spectra in methanol-d_4_ at 500 MHz of fusaric acid (**5**) (A), the fusaric acid-magnesium complex **2** (B), and the fusaric acid-iron complex **1** (C).

**Figure S6.** HRESIMS spectrum of compound **1.**

**Figure S7**. ^1^H NMR spectra of compound **4** (Acetone-d_6,_ 500 MHz)

**Figure S8**. HSQC spectra of compound **4** (Acetone-d_6,_ 500 MHz)

**Figure S9**. HMBC spectra of compound **4** (Acetone-d_6,_ 500 MHz)

**Figure S10** HRESIMS spectrum of compound **4.**

**Figure S11**. ^1^H NMR spectra of compound **7** (Methanol-d_4,_ 500 MHz)
